# Care Bundle to Improve Oxygen Maintenance and Events

**DOI:** 10.1097/pq9.0000000000000639

**Published:** 2023-03-13

**Authors:** Sandesh Shivananda, Sumesh Thomas, Sourabh Dutta, Christoph Fusch, Connie Williams, Kanekal Suresh Gautham

**Affiliations:** From the * Division of Neonatology, BC Women’s Hospital and Health Centre, University of British Columbia, Vancouver, British Columbia, Canada; † Section of Neonatology, Department of Pediatrics, South Health Campus, University of Calgary, Calgary, Alberta, Canada; ‡ Neonatal Division, Department of Pediatrics, Postgraduate Institute of Medical Education and Research (PGIMER), Chandigarh, India; § Department of Pediatrics, McMaster University, Hamilton, Ontario, Canada; ¶ Department of Pediatrics, General Hospital, Paracelsus Medical University, Nuremberg, Germany; ‖ Department of Pediatrics, University of Central Florida College of Medicine; ** Nemours Children’s Health System, Orlando, FL, USA.

## Abstract

Prolonged periods spent outside the target range of oxygen saturation (SpO_2_) in preterm infants, along with frequent desaturation events, predispose them to retinopathy of prematurity (ROP) and long-term neurodevelopmental impairment. The primary aim of this study was to increase the mean time spent within the target SpO_2_ range (WTR) by 10% and to reduce the frequency of desaturation events by 5 events per patient day, respectively, within 18 months of implementing a care bundle.

**Methods:**

This study was completed in a 46-bed neonatal intensive care unit (NICU), involving 246 staff members and led by a quality improvement team. The change interventions included implementing new practice guidelines, reviewing daily summaries of SpO_2_ maintenance, daily infant wellness assessment, standardizing workflow, and responding to SpO_2_ alarms. In addition, we collected staff satisfaction and compliance with change interventions, resource use, and morbidity and mortality data at discharge.

**Results:**

The mean time spent WTR increased from 65.3% to 75.3%, and the frequency of desaturation events decreased from 25.1 to 16.5 events per patient day, respectively, with a higher magnitude of benefit in infants on days with supplemental oxygen. Postimplementation, the duration of high-frequency ventilation and supplemental oxygen were lower, but morbidity and mortality rates were similar. Staff satisfaction with training workshops, coaching, use of the infant wellness assessment tool, and SpO_2_ alarm management algorithms were 74%, 82%, 80%, and 74%, respectively.

**Conclusion:**

Implementing a care bundle to improve oxygen maintenance and reduce desaturation events increased the time spent WTR and reduced the frequency of desaturation events.

## INTRODUCTION

Prolonged periods spent outside the target oxygen saturation (SpO_2_) range and frequent desaturations are associated with retinopathy of prematurity (ROP), bronchopulmonary dysplasia (BPD), mortality, and neurodevelopmental impairment.^1–7^ Factors implicated in frequent desaturations and maintaining appropriate saturation within the target SpO_2_ range (WTR) include immature breathing control, the severity of lung or other organ diseases, and variations in providers responding to SpO_2_ alarms.^8–11^ Recent evidence suggests that attaining a consistent optimal SpO_2_ range requires optimal use of technology and standardizing staff response to SpO_2_ alarms.^12,13^ SpO_2_ histograms and event review technology are standard features of bedside monitors to aggregate data over extended periods.^14^ It is the most common method used to measure the adequacy of SpO_2_ maintenance and frequency of desaturation events.^6,8–14^

### Problem Description

An audit in March 2014 within the McMaster Children’s Hospital neonatal intensive care unit (NICU) showed that, on average, in infants younger than 32 weeks gestation receiving supplemental oxygen, the SpO_2_ was WTR 46% of the time, and individual patients experienced 43 desaturation events daily. In contrast, other NICUs report maintenance of SpO_2_ within the target range 60%–66% of the time.^9–13^

A survey of McMaster NICU providers identified potential contributory factors, including incorrect alarm limit settings, inconsistent use of SpO_2_ event review features of the monitors, and lack of standardization of the responses to SpO_2_ alarms. Thus, our NICU had a significant opportunity for improvement.

In January 2014, we launched a quality improvement (QI) project in infants younger than 32 weeks gestation (1) to increase the mean SpO_2_ time spent WTR by 10% and (2) to reduce the frequency of desaturations by 5 events per patient day from baseline within 18 months. The secondary goals were to assess resource utilization and the discharge morbidity and mortality.

## METHODS

### Setting

We conducted this study in a 46-bed NICU with approximately 200 annual inborn and outborn admissions of infants younger than 32 weeks gestation and an average daily census of 43. We measure SpO_2_ using a Masimo SET Radical pulse oximeter (software version 46.02; Masimo Radical, Masimo Corporation) integrated into the bedside Intellivue monitor (Philips Healthcare Nederland).

### Baseline Process of SpO_2_ Maintenance and Event Management

Preintervention, the prescribed SpO_2_ alarm targets were 84%–93% for infants receiving supplemental oxygen and 84%–100% for infants not on oxygen supplementation. In addition, clinical teams did not routinely review SpO_2_ histograms and daily event review reports during bedside rounds. Thus, decisions about escalation or deescalation of oxygen therapy were subjective and primarily reflected the staff’s perception of oxygen requirements over the preceding 4–8 hours.

### QI Project

**Fig. 1.**
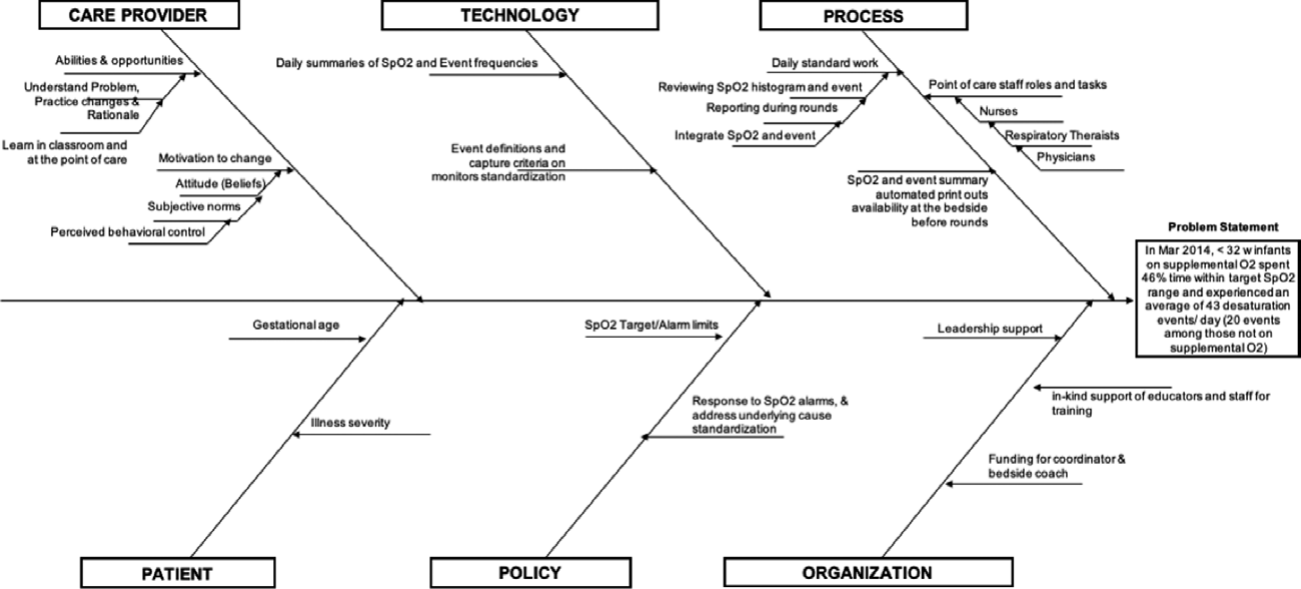
Cause-and-effect diagram. Understanding the challenges of SpO_2_ maintenance and frequent desaturations. O_2_ indicates oxygen; SpO_2_, saturation of oxygen.

**Fig. 2.**
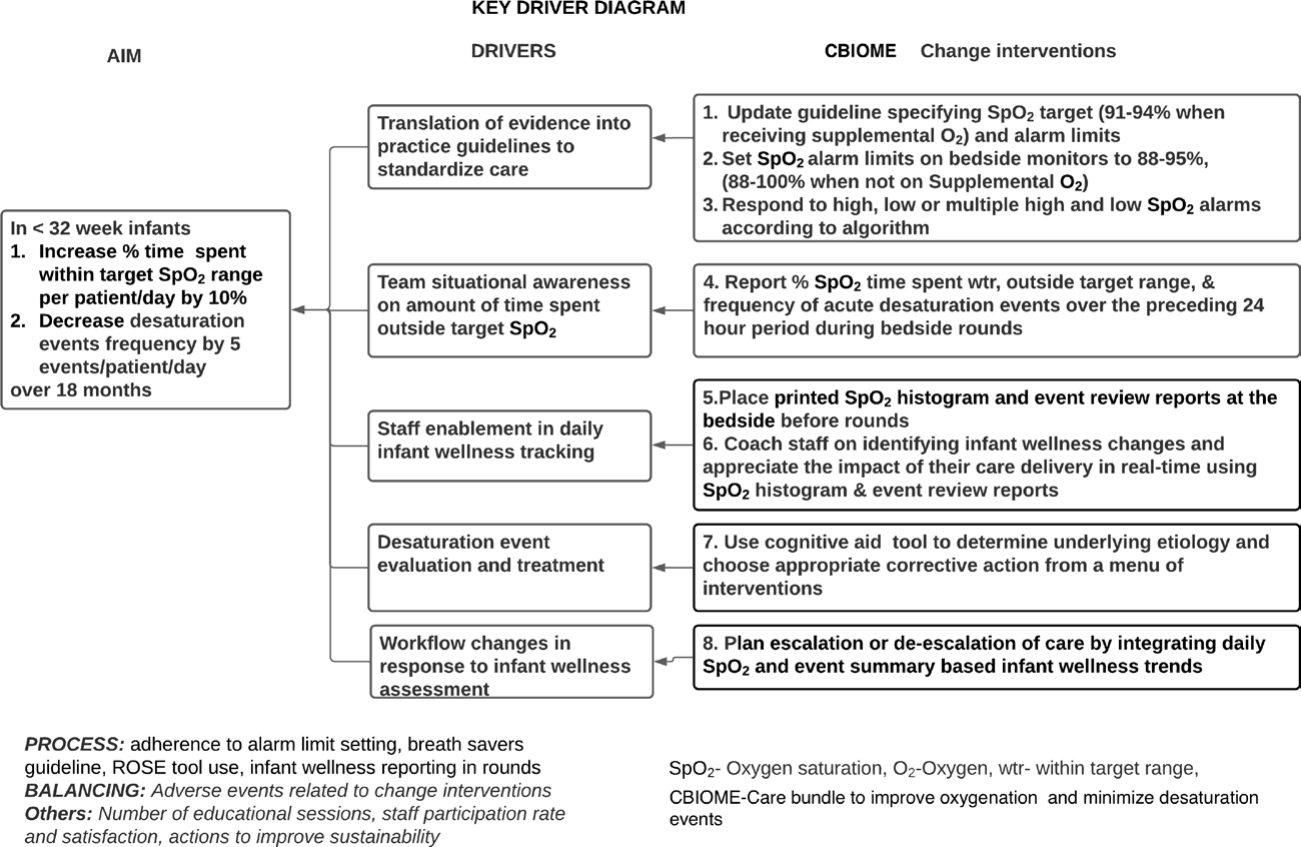
Key driver diagram to improve oxygen saturation maintenance and reduce desaturation events.

Our QI team included 2 physicians (MD), a nurse practitioner, a nurse educator, a respiratory therapy (RT) educator, an RT practice leader, a biomedical engineer, and a project facilitator. The team developed a project charter, deconstructed the problem using a cause-and-effect diagram (Fig. 1), and created a key driver diagram (Fig. 2). Interventions included new practice guidelines, reviewing daily summaries of SpO_2_ maintenance, daily infant wellness assessment, standardized workflow, and staff’s response to SpO_2_ alarms. Ten neonatologists, 20 fellows, 12 nurse practitioners, 160 nurses (registered nurse [RN]), 32 RTs, and 12 allied staff members participated in this study.

Study subjects included all infants admitted at younger than 32 weeks gestation but excluded infants with cyanotic congenital heart disease and other major congenital malformations. We monitored SpO_2_ and desaturation events from admission until discharge or until the infant breathed spontaneously without respiratory support or supplemental oxygen for more than 72 hours. We analyzed the data in 3 phases to simplify the scheduling of facilitators and the QI team members. These phases were preimplementation (May 12–November 30, 2014), implementation (December 1, 2014, to May 31, 2015), and postimplementation (June 1–December 31, 2015).

### Design and Testing of Changes to Improve SpO_2_ Targeting and Manage Events

**Fig. 3.**
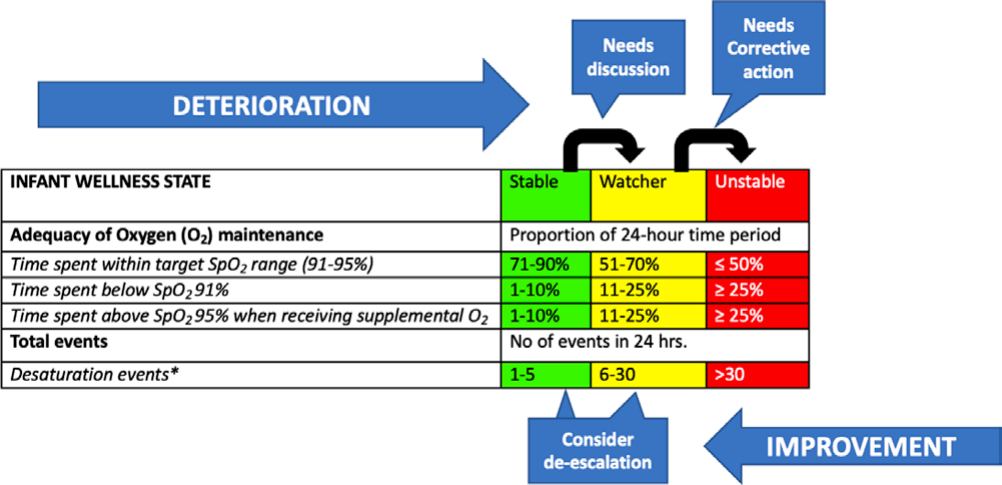
ROSE tool. *Desaturation event occurring in isolation or concurrently with bradycardia and/or apnea. The black arrow indicates a transition from one infant wellness state to another. Blue arrows indicate the trajectory of the infant wellness state. Callouts indicate suggested actions for care teams during bedside rounds. Needs discussion includes: discussing possible etiology, further workup, and optimizing supportive care. Needs corrective action includes elements under discussion plus (1) writing respiratory care plans in an order sheet, (2) discussing contingency plans for anticipated problems during rounds, and (3) sharing the plans with the evening team at handover. If an infant fits into the watcher and an unstable category under different wellness assessment elements, label infant wellness as “unstable”. This tool is for use on days with oxygen supplementation.

We hypothesized that effective implementation of evidence-based practices and technology integration would result in more time spent WTR with fewer desaturation events. A literature review identified appropriate SpO_2_ targets and alarm limits for preterm infants,^15–17^ algorithms to facilitate consistent response to SpO_2_ alarms,^13^ and strategies for implementation. We captured metrics relevant to the project by a daily review of the SpO_2_ histogram, event frequency, triggers for escalation, and weaning of care practices. These metrics were documented and tracked using a customized tool created for this project called the Review of Oxygen Saturation and Events (ROSE) tool (Fig. 3). Using this tool, we categorized an infant’s status into stable, watcher, and unstable states.

The care bundle to improve oxygenation and minimize desaturation events (Fig. 2) included:

1. unit guidelines specifying SpO_2_ target and alarm limits;
2. verification that alarm limits on the monitor corresponded to guidelines at the beginning of every shift;
3. standardization of staff response to SpO_2_ alarms (**Figure 1, Supplemental Digital Content, which describes guideline to standardize response to events by point of care staff,** http://links.lww.com/PQ9/A464);
4. reporting during daily rounds the percentage time spent within and outside the target SpO_2_ range and frequency of desaturations over the preceding 24-hour period (**Figure 2, Supplemental Digital Content,** which describes process map showing workflow changes and integration of CBIOME interventions into NICU daily routine and standard work, http://links.lww.com/PQ9/A465);
5. placement of printed SpO_2_ histogram and event review reports at the bedside
6. staff coaching to help them identify improvements or deteriorations based on daily SpO_2_ histogram and event review reports and appreciate the real-time impact of their care;
7. use of cognitive aids^18^ to determine underlying etiology for desaturation events and to choose corrective actions from a menu of interventions (**Table 1, Supplemental Digital Content,** which describes cognitive aid determining underlying etiology of frequent events or difficulty in oxygen saturation maintenance within desired SpO_2_ target range and potentially beneficial supportive care practices to improve infant’s stability, http://links.lww.com/PQ9/A467), and
8. systematic escalation or deescalation of care based on infant status.

### Implementation

All bedside monitors were configured to detect SpO_2_ data with a 20-second averaging time. We set alarm limit triggers for high and low SpO_2_ with a delay of 20 seconds and a 10-second delay for desaturation of 80 or lower. In addition, we set event capture triggers for a SpO_2_ less than 80 for 10 seconds, bradycardia of less than 80 per minute of any duration, and apnea of greater than 20 seconds.^16,19^ The monitors calculated the SpO_2_ histogram over 24 hours with a 1-second real-time sampling rate.

The QI team communicated changes via several formats (email, newsletter, and pocket cards) and forums (education days, professional group meetings, and seminars). All staff received training on setting appropriate SpO_2_ targets and alarm limits, responding to SpO_2_ alarms, and using the ROSE tool in a simulation-based interprofessional workshop, followed by bedside coaching (**Table 2, Supplemental Digital Content,** which describes NICU Team Workshop - CBIOME Facilitator Agenda, http://links.lww.com/PQ9/A468). We introduced interventions (Fig. 2) 1–5 in the first week of December 2014, interventions 6 and 8 in February 2015, and intervention 7 (**Table 1, Supplemental Digital Content,** which describes cognitive aid determining underlying etiology of frequent events or difficulty in oxygen saturation maintenance within desired SpO_2_ target range and potentially beneficial supportive care practices to improve infant’s stability, http://links.lww.com/PQ9/A467) in August 2015. Training workshops occurred between September and November 2014 (twice a week, half a day), and bedside coaching between February and June 2015. In addition, the QI team met monthly to review and address identified gaps and barriers to adoption.

In response to performance degradation in the postimplementation period, the QI team used specific interventions to enhance the staff’s compliance with the care bundle, such as bedside education, role modeling, defining standard work, and showing the benefit of infant wellness assessment using clinical vignettes. Other guidelines, respiratory and other supportive care practices, and nurse staffing did not change during the project. Moreover, the project coordinator daily reviewed the SpO_2_ histograms and events captured by the monitors and manually documented project metrics.

### Study of Interventions, Measures, and Outcomes

The primary outcomes were the proportion of time spent WTR and the frequency of desaturation events. These events occurred with or without bradycardia or apnea, as captured on the bedside monitor event report. We summed these measures for all patients and expressed them as a mean percentage of time spent WTR per patient day and the mean number of desaturation events patient day.

The secondary outcome measures were a mean percentage of time spent below and above the target SpO_2_ range (on supplemental O_2_ days) per patient day, the morbidity associated with the inadequacy of oxygen maintenance and frequency of desaturation (eg, BPD and ROP), mortality, the duration of ventilation, and hospital stay. We defined any day an infant received supplemental oxygen between 8 and 10 am as an oxygen supplemental day. We gathered outcomes at discharge from the unit’s database using standard definitions.^20^

The process measures observed during rounds were the target behaviors of RN, RT, and MDs, adherence to monitoring alarm limits, use of the SpO_2_ management algorithms, and the percentage of SpO_2_ time spent WTR. A QI nurse gathered the latter 2 process metrics in May and June 2015 by indirectly observing RN and RT responses to desaturation alarms and the providers’ presentations during rounds, respectively.^21^

The balancing measures were adverse events associated with the care bundle (eg, an event requiring intubation or chest compression secondary to a delayed response or seizures) captured from an existing adverse event reporting system and adjudicated by a quality assurance nurse. Furthermore, we documented the frequency of the education sessions and staff participation, measured staff satisfaction using validated questionnaires,^22^ and identified the steps required to sustain changes.

### Analysis

We used data from all patient days to determine the mean percentage of time spent per patient day within and outside the target SpO_2_ range and the desaturation frequency. To determine the mean percentage of time spent above the target SpO_2_ range, we used only patient days on oxygen supplementation. We analyzed the change over time using p- and u-charts (created using QI Macros for excel 2018, KnowWare International, Inc., Denver, CO) by plotting data weekly and identifying special cause variation using standard rules.^23,24^ We compared infant characteristics and outcomes at discharge during 3 phases of implementation. The change in observed target behaviors was analyzed using the Chi-square or Fischer exact tests and Kruskal-Wallis test (IBM Corp. Released 2020. IBM SPSS Statistics for Macintosh, Version 27.0. Armonk, NY: IBM Corp).

### Ethical Consideration

The hospital ethics board deemed this project a QI initiative and exempted the study from full ethics board review. The QI team adhered to the hospital’s privacy policy.

## RESULTS

### Demographics

During the study, we included 329 infants with a median (interquartile range) gestational age and birth weight of 28 weeks (26−30) and 1160 g (812−1452), respectively. The demographic profile of infants in the 3 phases was similar (Table 1). There were 10,453 patient days of SpO_2_ histogram and event report data after excluding 3122 patient days of missing data (23%). In addition, there were 6003 patient days when infants received supplemental oxygen. The NICU bed occupancy rate ranged from 86% to 100%, with no difference between the 3 study phases.

**Table 1.** Demographic Profile of Infants at Admission

Parameter	Preimplementation Phase (May–November 2014) N = 120 Infants	Implementation Phase (December–May 2015) N = 102 infants	Postimplementation (Jun–December 2015) N = 107 infants	*P*
Gestational age, weeks*	28 (26−31)	29 (27−30)	28 (27−30)	0.48
Birth weight, g*	1120 (812−1515)	1185 (915−1465)	1183 (910−1452)	0.89
Male gender	68 (56.7)	57 (55.9)	56 (52.3)	0.78
Outborn	11 (9.2)	18 (17.6)	19 (17.8)	0.10
Antenatal steroids received	111 (92.5)	92 (90.2)	94 (87.9)	0.37
Rupture of membrane, h	24 (20.0)	22 (21.6)	30 (28.0)	0.36
C section delivery	69 (57.5)	63 (61.8)	65 (60.7)	0.79
Apgar 1 min*	5 (4−7)	6 (4−8)	6 (4−7)	0.17
Apgar 5 min*	8 (7−9)	8 (7−9)	8 (7−9)	0.66
PPV by bag and mask or T-piece resuscitator at birth	81 (67.5)	70 (68.6)	80 (74.8)	0.44
PPV by endotracheal tube at birth	30 (25.0)	26 (25.5)	34 (31.8)	0.45
Chest compression at birth	10 (8.3)	6 (5.9)	9 (8.4)	0.73
Snappe II*	14 (5−34)	14 (6−31)	18 (9−31)	0.52

Data in each phase are not randomized.

*Median (interquartile range), values in cell represent n (% unless stated otherwise). *P* value <0.05 is significant.

PPV, positive pressure ventilation, SNAPE II, Score for Neonatal Acute Physiology with Perinatal extension-II.

### Time Spent WTR and Frequency of Desaturation Events of the Entire Study Population

**Fig. 4.**
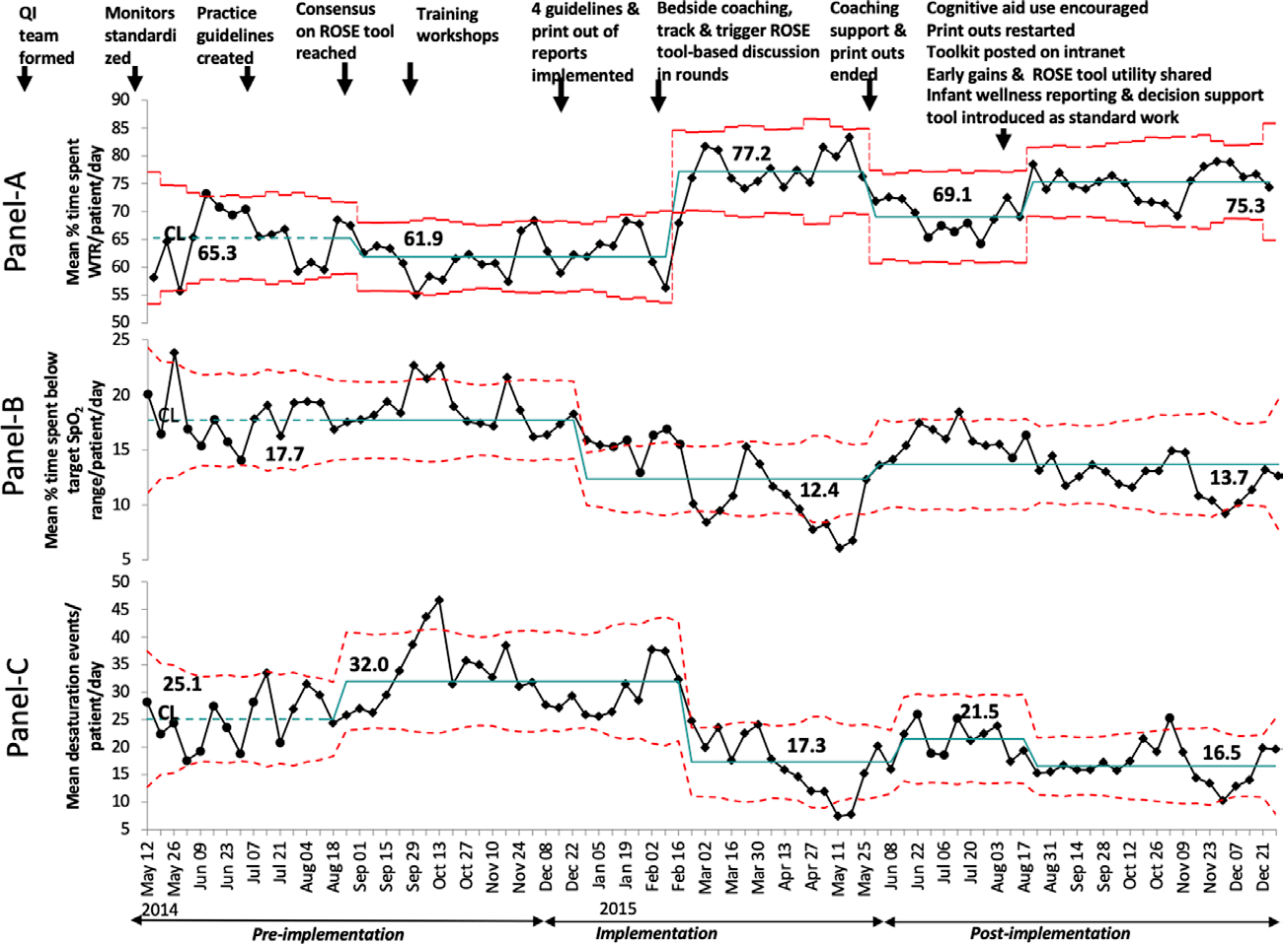
Control charts showing the mean percentage of time spent within and below the target SpO_2_ range and mean frequency of desaturation events per patient per day. Annotated p-charts (A) and (B) and u-chart (C). Each dot represents the mean % of time spent within the target SpO_2_ range and below the target SpO_2_ range per day by all patients in that week and the mean desaturation events per day by all patients in that week. Diamond and circle-shaped dots represent unstable points (out-of-control process) and stable points, respectively. Centerline shifts conformed to standard statistical process control rules (8 points in a row). Dotted lines indicate upper and lower control limits.

The mean percentage of time spent WTR per patient day increased from a baseline of 65.3%–75.3% (Fig. 4A). The mean desaturation frequency decreased from a baseline of 25.1 to 16.5 events per patient day (Fig. 4C). Special cause variation with centerline shifts for the mean percentage of time spent WTR; (Fig. 4A) and frequency of desaturation events (Fig. 4C) occurred on 4 occasions at the beginning of 2014-August 25, 2015-February 9, May 18, and August 17. The mean percentage of time spent below the target SpO_2_ range (Fig. 4B) decreased from a baseline of 17.7% to 13.7%, with centerline shifts occurring on 2 occasions; December 22, 2014, and May 25, 2015.

**Fig. 5.**
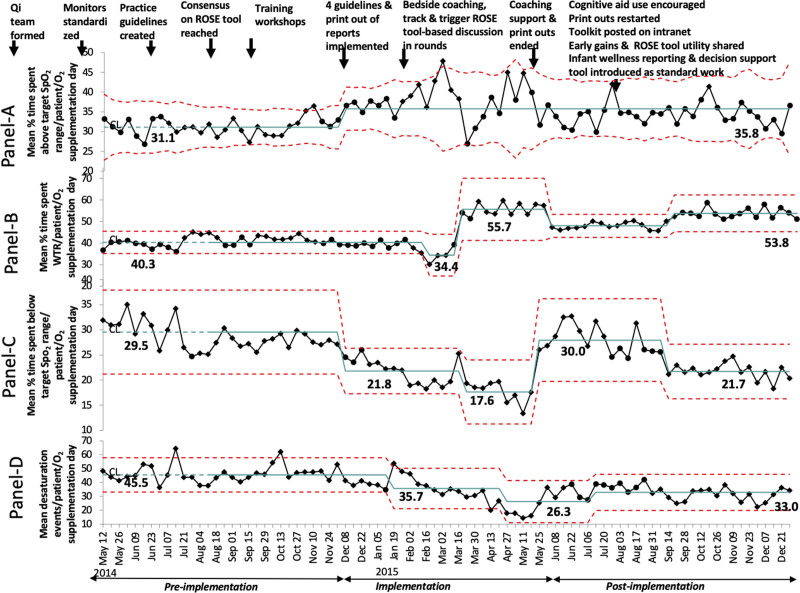
Control charts showing the mean percentage of time spent above, within, and below the target SpO_2_ range and mean frequency of desaturation events per patient per oxygen supplementation day during the study. Annotated p-charts (A)–(C) and u-chart (D). Each dot represents the mean % of time spent above the SpO_2_ target range, within the target SpO_2_ range, and below the target SpO_2_ range per oxygen supplementation day by all patients in that week (A)–(C), respectively, and mean desaturation events per oxygen supplementation day by all patients in that week (D). Diamond and circle-shaped dots represent unstable points (out-of-control process) and stable points, respectively. Centerline shifts conformed to standard statistical process control rules (8 points in a row). Dotted lines indicate upper and lower control limits.

Subgroup analysis of infants on supplemental oxygen (Fig. 5A–D) reveals centerline shifts on 4 occasions for the mean percentage of time spent WTR and below the target SpO_2_ range (Fig. 5B, C) respectively; 3 occasions for frequency of desaturation event (Fig. 5D) and on 1 occasion for a mean percentage of time spent above target SpO_2_ range (Fig. 5A). On days without supplemental oxygen, we noted centerline shifts on a few occasions (**Figure 3, Supplemental Digital Content,** http://links.lww.com/PQ9/A466), but the magnitude of benefit was minimal.

### Resource Use, Morbidity, and Mortality During the NICU Stay

The proportion of infants receiving various treatments, such as inotropes and antibiotics during the 3 phases (preimplementation, implementation, and postimplementation) was similar, except for the use of noninvasive positive pressure ventilation (**Table 3, Supplemental Digital Content,** which describes treatment received by infants during NICU stay, http://links.lww.com/PQ9/A469). The duration of high-frequency ventilation, need for intubation and ventilation, use of supplemental oxygen, and antibiotic days per 100 NICU patient days, unadjusted for covariables, were significantly lower in the postimplementation period (Table 2) (**Table 2, Supplemental Digital Content,** which describes NICU Team Workshop - CBIOME Facilitator Agenda, http://links.lww.com/PQ9/A468). There was no difference in morbidity and mortality rate (**Table 4, Supplemental Digital Content,** which describes infant outcomes at NICU discharge, http://links.lww.com/PQ9/A470)

**Table 2.** Duration of Treatment Received by Infants

Treatment intervention	Preimplementation Phase (May–November 2014) N = 120 Infants. Days per 100 NICU Patient Days	Implementation Phase (December–May 2015) N = 102 Infants. Days per 100 NICU Patient Days	Postimplementation (June–December 2015) N = 107 Infants. Days per 100 NICU Patient Days	*P*
High-frequency ventilation days	13.1	7.8	8.0	0.0001
Intubation and ventilation days	8.0	8.9	5.1	0.0001
Noninvasive positive pressure ventilation days	6.7	5.6	4.2	0.0001
Continuous positive airway pressure ventilation days	40.7	45.6	61.8	0.0001
High flow days	13.0	11.0	8.9	0.0001
Supplemental oxygen days	58.2	41.6	48.0	0.0001
Caffeine days	66.7	64.7	72.7	0.0001
Antibiotic days	30.1	26.7	23.6	0.0001

Data in each phase are not randomized.

Values in cell represent intervention days per 100 NICU patient days. *P* value < 0.05 is significant (Chi-Square test).

### Process Measures

The frequency of appropriately set alarm limits increased from 80% in the preimplementation phase to 95% in the postimplementation phase. Bedside printouts were available on all days in the implementation and postimplementation periods, except for 3 weeks in June 2015. Staff used the ROSE tool and discussed findings during rounds in 499 (70%) out of 714 patient day encounters when assessed in May and June 2015. Two hundred thirty-four care providers attended the workshops, 160 received bedside coaching, and 90 attended 9 meetings or sessions on reinforcing practices. Staff compliance with SpO_2_ alarm management algorithms increased from a baseline of 0% (September 2014) to 64% during May and June 2015. In addition, the staff (n = 63, 53%) reported “satisfied/highly satisfied” with training workshops, bedside coaching, ROSE tool use, and SpO_2_ alarm management algorithms 74%, 82%, 80%, and 74%, respectively.^21^ Suggested steps to sustain changes were transitioning to automated assessment of infant wellness and validating the ROSE tool with meaningful outcomes like escalated care events or morbidity at discharge.

### Balancing Measures

Staff reported no care bundle-related patient safety incidents in the implementation or postimplementation phases.

## DISCUSSION

Our QI initiative was associated with an observed increase in the mean duration of time spent within the target range of oxygen saturation and a reduction in the frequency of desaturation events. This overall improvement occurred despite degradation on 2 occasions. This change was initially due to better compliance with the older guideline’s wider alarm limits (84%−93%) and later because of a temporary discontinuation of histogram reports to conserve paper at the beginning of the postimplementation period (Fig. 4A, C). We successfully integrated a multicomponent care bundle into the routine practices of our NICU with no negative impact on patient outcomes.

Among the multiple interventions, the key interventions that are likely to have led to the observed improvements, based on temporal association (Figs. 4, 5), are the introduction of guidelines, bedside availability of histogram and event review reports, coaching, and standardized reporting of infant wellness using the ROSE tool. In addition, we observed a higher magnitude of improvement on days with supplemental oxygen (Fig. 5; **Figure 3, Supplemental Digital Content,** which describes control charts showing the mean percentage of time spent above, within, and below the target SpO_2_ range and mean frequency of desaturation events per patient on days without oxygen supplementation during the study, http://links.lww.com/PQ9/A466). We speculate this because this subgroup had greater opportunities for easy improvements (“low-hanging fruit”), with a higher frequency of oxygen saturation values outside the desired range and a higher rate of desaturation events.

We encountered 3 important challenges during this project. The first was choosing the SpO_2_ target range for infants 28–31 weeks. The published evidence is limited. To maintain uniformity of practice and offer the benefits of narrow alarm limits, we chose the same SpO_2_ targets for infants born between 28 and 31 weeks as infants of less than younger than 28 weeks.^9,15–17^

The second challenge was the staff’s dependence on printed reports. A printed report eased staff workload, provided a physical cue to perform their tasks (ie, plotting and reporting), and made it easier to track improvement in the infant status.^25^ Thus, we continued the printouts until the end of the project with a plan to automate the ROSE tool-based infant wellness categorization and displaying it on bedside monitors in the future.

Finally, the staff were skeptical about using the ROSE tool, as it might lead to undertreatment or overtreatment. We addressed this skepticism by educating staff about the ROSE tool and emphasizing that it was an aid to, but not a substitute for, their bedside clinical judgment and decision-making about the use of supplemental oxygen and respiratory care management.

How do the results of our study compare with those of others? Such a comparison is hampered because other studies differed in some aspects of their methodology. There were differences in contexts, definitions (eg, maintenance of oxygen saturation in the target range), inclusion criteria, and duration of observation. Some aspects of our data, specifically the 14% increase in time spent WTR and 36% time spent above the SpO_2_ target range in infants on oxygen supplementation, are comparable to other studies aimed at improving oxygen maintenance.^12,13^ However, other studies did not observe a reduction in time spent below the SpO_2_ target range.^12,13^

A worrisome observation in our project in all phases was the high percentage of time the SpO_2_ was above the target range (30%−35%) in infants receiving oxygen supplementation during all phases. This finding highlights the challenges and limitations of manual bedside adjustment of supplemental oxygen by nurses or respiratory therapists. Emerging research suggests that we can maintain SpO_2_ in the target range more frequently with automated closed loop-controlled oxygen administration than with human-based systems.^26,27^

Although previous studies describe the frequency of desaturation events and their natural progression with postnatal age,^19,28–30^ our study is the first to show the impact of interventions on the frequency of desaturation (Fig. 5). We did not observe any change in ROP or mortality by choosing a higher SpO_2_ target range, in contrast to the NeOProM study results.^31^ However, that study was based on randomized controlled trial data and is considered a higher level of evidence than our study. Similarly, our study did not change BPD rates, possibly because the care bundle did not include many practices associated with reducing BPD.^32,33^ We speculate that standardized monitoring of infant stability and early extubation and/or weaning oxygen may have contributed to a reduction in invasive ventilation and supplemental oxygen days observed in this study. Our study’s novel interventions were improved communication, staff ownership,^34^ workflow integration,^35^ and the use of readily available daily summaries from monitors with no sophisticated custom data acquisition or analysis. Thus, these interventions are generalizable to most neonatal units.

This study had several limitations, many of which are innate limitations of pragmatic QI projects.

1. We introduced the first 5 changes simultaneously as a care bundle and not in a stepwise fashion. Therefore, we cannot estimate the magnitude of the impact of individual change interventions. In addition, the ROSE tool we used to improve communication and identify changes in infant wellness is an unvalidated tool.
2. Survey responses are subject to response bias.
3. Although we monitored for adverse events, we might have missed such events that were infrequent or unreported.
4. Inability to rationally subgroup infants according to illness severity resulted in several unexplained unstable points on control charts.
5. The lack of resources prevented us from gathering data on weekends (23% missing data), and certain detailed data, such as the depth and duration of each apnea, bradycardia, desaturation, or rebound hyperoxia events; the frequency of use of the cognitive-aid tool and its utility in escalation or deescalation of care.

Although in the absence of an experimental study design, we cannot be completely certain that the observed changes resulted from concurrent changes in patient acuity, unit census, nurse staffing ratios, and other care practices. Nevertheless, we believe that the observed gains resulted from our change package.

## CONCLUSIONS

Implementing a care bundle intended to improve the management of SpO_2_ in preterm infants is associated with increased time spent within the desired target SpO_2_ range and decreased frequency of desaturation events. In addition, using care bundles can standardize practices, facilitate technology integration, and enable the staff to adopt new practices in their daily routine.

## ACKNOWLEDGMENTS

We thank all the QI team members (Middleton K, Rich B, Dilario J, Deb B, Paterson D, Dyck G, Schattauer K, and Pogorzelski D) for facilitating training, change management, and data collection. We thank all the neonatologists for their input in designing and implementing change interventions, especially Helou S, Zafarghandy S, Wahab MG, El Gouhary E, Marrin M, Meyer CL, Newbube A, Shah J, and Pugh E. Finally, we thank all house staff, charge nurses, practice leaders, educators, managers, and charge respiratory therapists for being engaged as early adopters and working through their change process, leading change process within their teams, and supporting reinforcement mechanisms, including audits, compliance measuring, ongoing training, and coaching.

## DISCLOSURE

The authors have no financial interest to declare in relation to the content of this article.

## Supplementary Material


