# Supplementary material for: Care Bundle to Improve Oxygen Maintenance and Events
Source: Pediatr Qual Saf. 2023 Mar 13;8(2):e639. doi: 10.1097/pq9.0000000000000639 (PMC10013622; doi:10.1097/pq9.0000000000000639)
Supplement: Supplementary file 1 [file pqs-8-e639-s001.pdf]

A

Troubleshooting Causes of Events (Desaturation/Apnea/Bradycardia)

The 5 P's mnemonic provides a universal approach to identifying and intervening in the most common event causes, allowing for such causes to be immediately corrected.

The 5 P's include:

- ✓ Positioning
- ✓ Prongs
- ✓ Patency of the airway
- ✓ Probe
- ✓ Physical Assessment

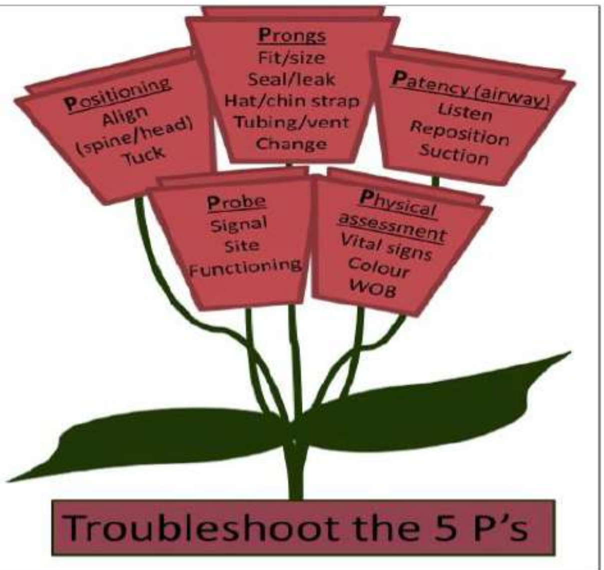

B

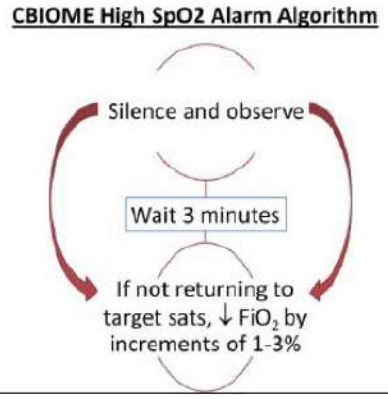

- For infants who are experiencing a high SpO2 alarm, we will silence the alarm and observe.
- After waiting 3 minutes, we reassess the SpO2 level and if not returning to target saturations, we will decrease the FiO2 by 1-3% and silence and observe for an additional 3 minutes.
- We will repeat this cycle as needed, in order to reach the target saturation range for our patient.

C

CBIOME Low SpO2 Alarm Algorithm

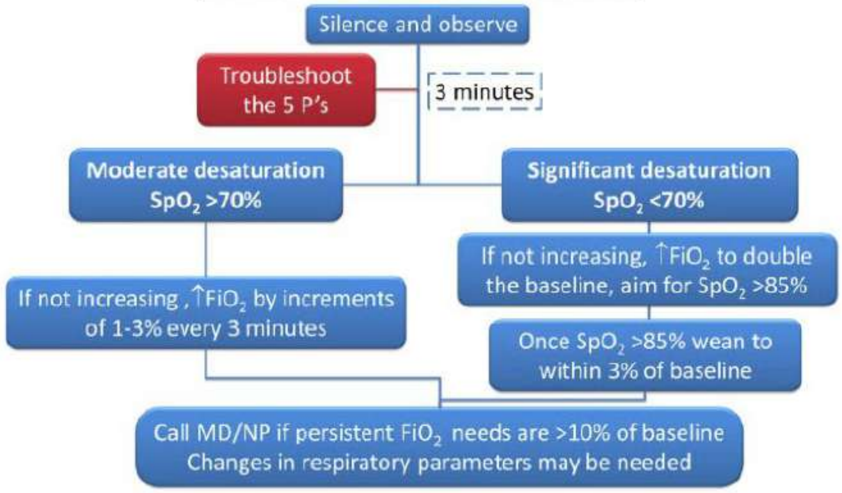

- For infants who are experiencing a desaturation event, our initial action will be to silence the alarm.
- After silencing the alarm, we will respond by troubleshooting potential causes for the event using the "5 P's" mnemonic (see above).
- At the 3 minute mark, we will classify the desaturation as either moderate or significant and respond by increasing FiO2 according to the algorithm.

D

CBIOME Multiple High and Low SpO2 Alarms Algorithm

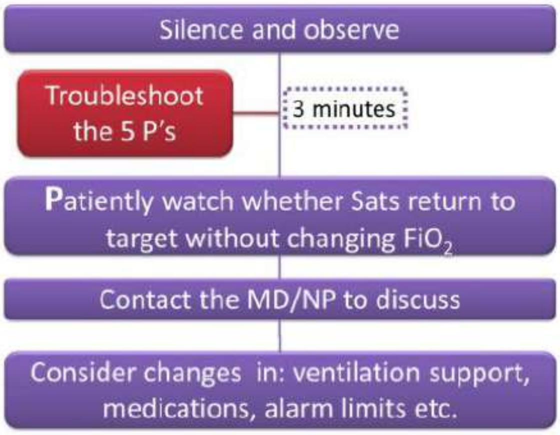

- Infants who are experiencing multiple high and low SpO2 alarms pose additional challenges that require a collaborative approach.
- For infants who are experiencing multiple high and low SpO2 alarms, our initial action will be to silence the alarm.
- After silencing the alarm, we will respond by troubleshooting potential causes for the event using the "5 P's" mnemonic (see above).
- Do your best to watch patiently, avoiding unnecessary adjustments in FiO2.
- Contact MD/NP to discuss and consider changes appropriate to the patient's needs.[+]
