# Supplementary figures and images for: Care Bundle to Improve Oxygen Maintenance and Events
Source: Pediatr Qual Saf. 2023 Mar 13;8(2):e639. doi: 10.1097/pq9.0000000000000639 (PMC10013622; doi:10.1097/pq9.0000000000000639)

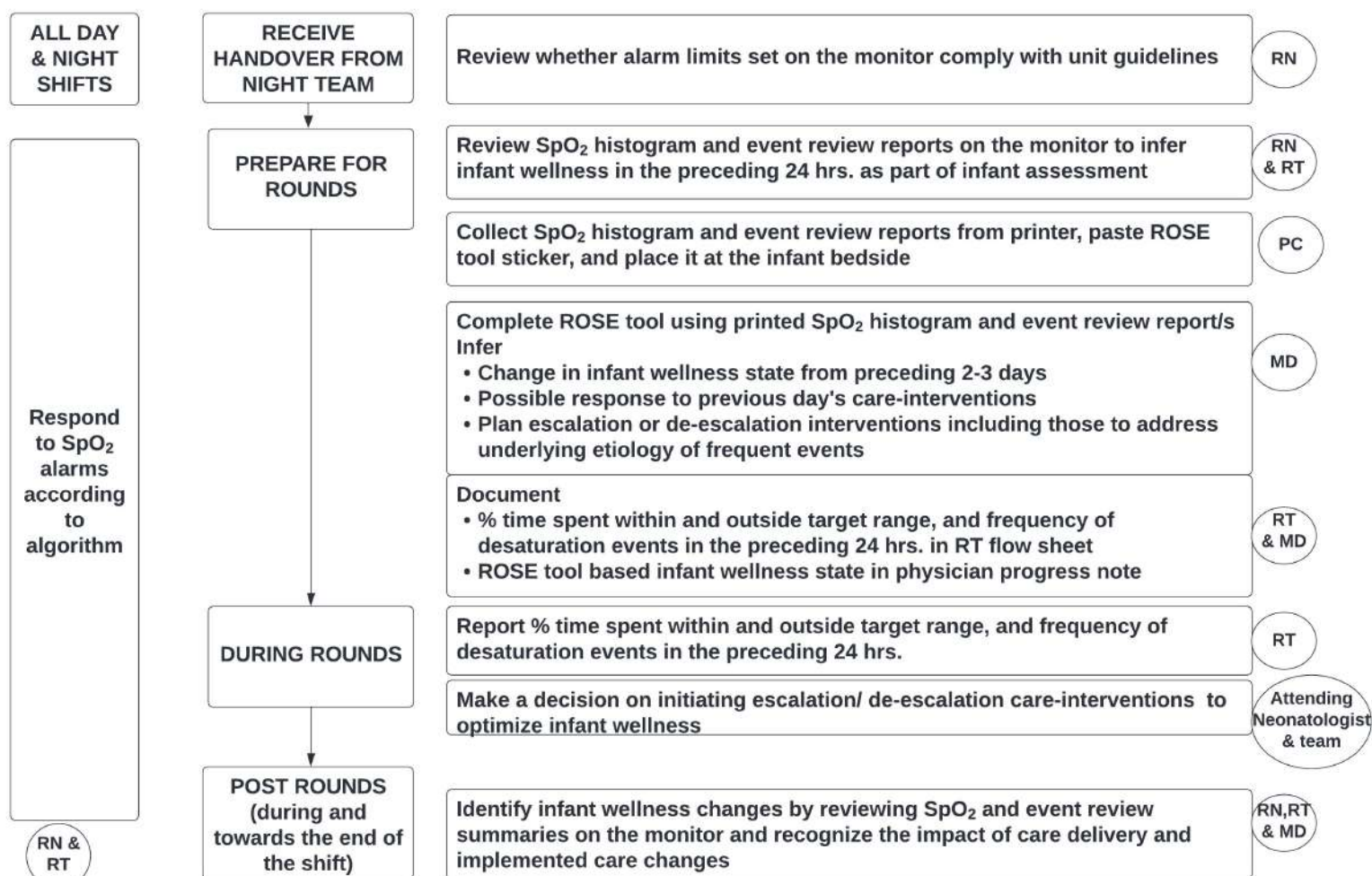

Supplement: Supplementary file 2 [file pqs-8-e639-s002.pdf]

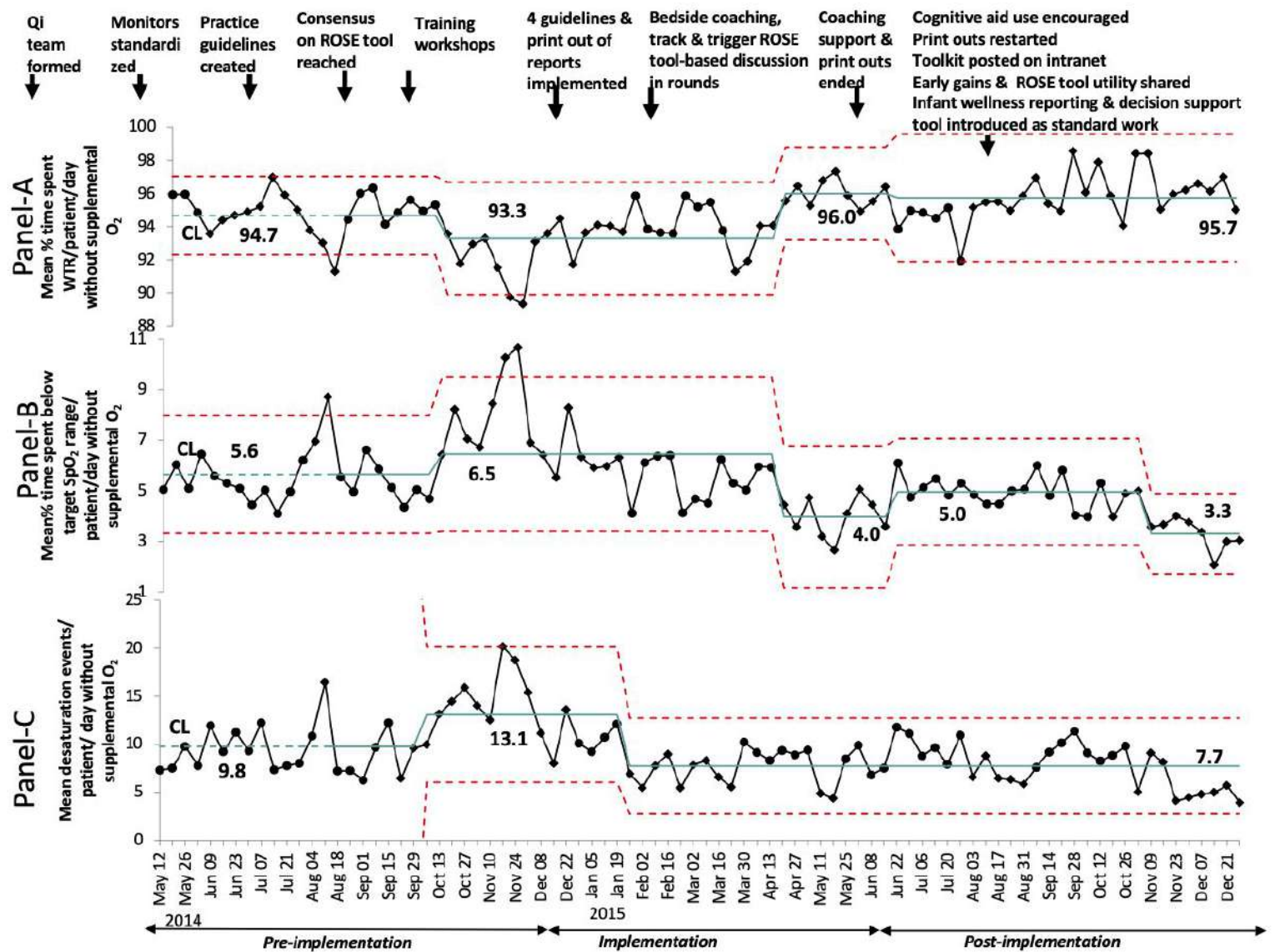

Supplement: Supplementary file 5 [file pqs-8-e639-s005.pdf]
