# Supplementary material for: Care Bundle to Improve Oxygen Maintenance and Events
Source: Pediatr Qual Saf. 2023 Mar 13;8(2):e639. doi: 10.1097/pq9.0000000000000639 (PMC10013622; doi:10.1097/pq9.0000000000000639)
Supplement: Supplementary file 3 [file pqs-8-e639-s003.pdf]

Supplementary Table 1: Cognitive aid determining underlying etiology of frequent events or difficulty in oxygen saturation maintenance within desired SpO2 target range and potentially beneficial supportive care practices to improve infant's stability

Etiology and management considerations in an infant with difficulty in oxygen saturation maintenance and /or frequent events (RED on ROSE tool)

Common causes for instability Caregivers should review the list to determine whether some of these factors are responsible for instability in a particular infant

Modifiable factors

Very common

- Mechanical issues (positioning of infant, water in hose, ill fitting prongs/masks/ interface, small size ET tube, accidental extubation, clearing of secretions, nasal passage obstruction due to clogged secretions/blood, open mouth, equipment malfunction, distended stomach, NG/OG tube in esophagus, Constipation)
- Airway obstruction- No air entry/breath sounds on auscultation airway obstruction
- Fighting on ventilator
- Excessive handling
- Irritable infant (due for feeding, diaper change, nasal excoriation, insecure ET tube)

Common

- Inadequate recruitment or respiratory drive,
- High ventilator pressures

Others

- Fluid overload and pulmonary edema (PDA, BPD)
- Pulmonary complications; pneumothorax, hemorrhage, effusion, abscess, collapse
- GERD- Reflux
- Infections (bacterial, viral, mycoplasma, fungal)
- Metabolic bone disease with excessively compliant chest wall
- Brain injury with abnormal spontaneous respiratory drive, Seizures
- Sedation or procedures (ROP screening, PICC line insertion, Immunization)
- Airway problems (vocal cord edema, laryngomalacia, bronchomalacia, vocal cord palsy)
- Neuromuscular disorders or syndromes

Non-modifiable factors

- Underlying severe or complicated respiratory, cardiac, neurological, GI, syndrome or other conditions

Potentially beneficial supportive care practices that may improve infant's stability

One or more of the following interventions can be applied based on individual needs of an infant. Every intervention should be evaluated after applying on an infant.

Respiratory practices

- Auscultating for air entry on chest to ensure CPAP/HF is being delivered
- Developmentally supportive care positioning, prone positioning, skin to skin care
- Chin strap
- Avoiding frequent handling
- Changing to appropriate type and size of nasal interfaces, ET tube
- Confirming NG tip placement
- Clearing nostrils and back of nose
- Venting of stomach
- Preventing water logging in the hose
- Appropriate ventilator, mode of ventilation and optimal lung recruitment
- Weaning based on integrated assessment (clinical features, gases, histogram, natural history) and reviewing response to previous weaning
- Optimizing Caffeine dose

Non-respiratory practices

- Fluid restriction/Optimizing total fluid intake (TFI)
- Diuretics
- Slow bolus feeds
- Blood transfusion
- Medical or surgical treatment of PDA
- Optimizing nutrition
- Ruling out viral, fungal or mycoplasma pneumonia by sending nasal secretions for workup
- Flexible nasal laryngoscopy
- Ruling our structural cardiac disease or cardiac dysfunction on an echo

Shivananda S for CBIOME program, McMaster Children's Hospital, Jan 2015

Footnote ET-endotracheal, NG-nasogastric, OG-orogastric, PDA-Patent ductus arteriosus, BPD-Bronchopulmonary dysplasia, GERD- Gastroesophageal reflux disease, ROP-Retinopathy of prematurity, PICC-Percutaneously inserted central catheter, GI- Gastro intestinal, CPAP-Continuous positive airway pressure, HF-high flow.
