# Supplementary material for: Care Bundle to Improve Oxygen Maintenance and Events
Source: Pediatr Qual Saf. 2023 Mar 13;8(2):e639. doi: 10.1097/pq9.0000000000000639 (PMC10013622; doi:10.1097/pq9.0000000000000639)
Supplement: Supplementary file 4 [file pqs-8-e639-s004.pdf]

Supplementary Table 2
NICU Team Workshop - CBIOME Facilitator Agenda

Time	***Topic***	***Details***
5 min.	Welcome and Overview	Details
25 min.	Questions and Answers from pre-reading CBIOME Fact Sheet	• Solicit questions from participants<br>• "Do you have any questions from the pre-reading you did?<br>• "Is there anything that is not clear to you at this point?""Is there anything that you are wondering about?"<br>• Once you have the list of questions, answer them (ideally by group discussion) or defer them (if they will be specifically addressed later in the workshop)
30 min.	CBIOME Jeopardy	• Divide participants into two teams (ideally with equal representation fromdisciplines represented)<br>• Play Jeopardy game to reinforce key concepts and components
60 min.	Case Simulations	• Ideally 4:1 (Participant: Facilitator)<br>• 4 cases (team members each take the "lead" in 1 case)<br>Cases are 5 minutes of simulation with 10 minutes of debriefing/discussionCases are:<br>o Moderate Desaturation (Preoxygenation scenario)<br>o Significant Desaturation (Troubleshoot the 4 Ps)<br>o Multiple High and low Alarms (Swinger scenario)<br>o High Alarm Condition (BPD)
15 min.	Break	
30 min.	Using Oxygen Histogram and Event Reports to Optimize Care	• Didactic – 10 min.<br>• Paper-based case study – individually completed – 10 min.<br>Group discussion of the case study – 10 min.
30 min.	Effective Communication for CBIOME	• Didactic Session with group discussion<br>• Report Practice in pairs (or) Video – Inadequate vs. Optimal Report
30 min.	Workshop Debriefing and Evaluation	• Refer back to questions collected at the start of the workshop and confirm withparticipants that all of the questions have been addressed. If not, address them now.<br>• Solidify learning by debriefing class as a whole. Ask participants to consider and then share (if comfortable) their own biggest "take away" or "key lesson" that they will integrate into their practice.<br>• Personal declaration of commitment to our patients (card to fill out and sign)<br>Have participants complete a workshop evaluation form.
15 min.	Course Ends	

CBIOME- care bundle to improve oxygen maintenance and reduce events, BPD- Bronchopulmonary dysplasia

Bedside coaching details

Time	*Focus*	*Details*
5-15 mins per staff	Consolidation of skills on adopting practice changes and use of tools	Observe staff response to SpO_2 alarms using SpO_2 alarm management guideline, debrief their actions, encourage them sharing their experience
	Motivate staff in adopting practices consistently into their workflow	Help staff understand the reasons behind practice changes, help them overcome challenges preventing them from adopting the changes, and share details on how peers are adopting new practices as new norm
