# Supplementary material for: Care Bundle to Improve Oxygen Maintenance and Events
Source: Pediatr Qual Saf. 2023 Mar 13;8(2):e639. doi: 10.1097/pq9.0000000000000639 (PMC10013622; doi:10.1097/pq9.0000000000000639)
Supplement: Supplementary file 6 [file pqs-8-e639-s006.pdf]

Supplementary Table 3: Treatment received by infants during NICU stay

Treatment intervention	Pre-implementation phase (May – Nov 2014) N=120 infants	Implementation phase (Dec – May 2015) N=102 infants	Post-implementation (Jun – Dec 2015) N = 107 infants	P value
Inotropes	21 (17.5)	14 (13.7)	13 (12.1)	0.50
Postnatal systemic steroids for any indication	18 (15.0)	13 (12.7)	16 (15.0)	0.86
Postnatal systemic steroids for BPD prevention or treatment	13 (10.8)	6 (5.9)	6 (5.6)	0.24
Inhaled nitric oxide	6 (5.0)	4 (3.9)	8 (7.5)	0.50
Surfactant	58 (48.3)	45 (44.1)	63 (58.9)	0.08
Peripheral intravenous catheter	110 (91.7)	88 (86.3)	98 (91.6)	0.32
Umbilical arterial catheter	39 (32.5)	23 (22.5)	39 (36.4)	0.08
Umbilical venous catheter	76 (63.3)	57 (55.9)	72 (67.3)	0.22
Percutaneously inserted central catheter	53 (66.2)	44 (43.1)	62 (57.9)	0.052
High frequency ventilation	36 (30.0)	25 (24.5)	31 (29.0)	0.63
Intubation and ventilation	56 (46.7)	42 (41.2)	59 (55.1)	0.12
Non-invasive positive pressure ventilation	41 (34.2)	18 (17.6)	32 (29.9)	0.01
Continuous positive airway pressure	113 (94.2)	93 (91.2)	101 (94.4)	0.58
High flow	44 (36.7)	34 (33.3)	33 (30.8)	0.64
Supplemental Oxygen	100 (83.3)	84 (82.4)	94 (87.9)	0.49
Caffeine	103 (85.8)	87 (85.3)	96 (89.7)	0.57
Antibiotics	101 (84.2)	84 (82.4)	94 (87.9)	0.52
Blood products	49 (40.8)	29 (28.4)	43 (40.2)	0.10

(Data in each phase is not randomized). Values in cell represent n (% unless stated otherwise). BPD- Bronchopulmonary dysplasia. P value < 0.05 is significant
