# Supplementary material for: Care Bundle to Improve Oxygen Maintenance and Events
Source: Pediatr Qual Saf. 2023 Mar 13;8(2):e639. doi: 10.1097/pq9.0000000000000639 (PMC10013622; doi:10.1097/pq9.0000000000000639)
Supplement: Supplementary file 7 [file pqs-8-e639-s007.pdf]

Supplementary Table 4: Infant outcomes at NICU discharge

	Pre-implementation phase (May – Nov 2014) N=120 infants	Implementation phase (Dec – May 2015) N=102 infants	Post-implementation (Jun – Dec 2015) N = 107 infants	P value
Death	8 (6.7)	9 (8.8)	5 (4.7)	0.48
Length of stay among survivors, days median (IQR)	28 (8-74)	19 (10-56)	41 (14 -67)	0.129
Retinopathy of prematurity ≥ Stage 3	8 (6.7)	7 (6.9)	2 (1.9)	0.17
Treated for Retinopathy of prematurity [1]	4 (3.3)	3 (2.9)	0 (0)	0.17
Nosocomial infection [2]	20 (16.7)	12 (11.8)	15 (14.0)	0.57
Necrotizing enterocolitis ≥ Stage 2	7 (5.8)	7 (6.9)	4 (3.7)	0.59
Neuroimaging abnormal findings [3]	13 (10.8)	8 (7.8)	16 (15.0)	0.26
Treated for patent ductus arteriosus [4]	24 (20.0)	19 (18.6)	23 (21.5)	0.87
Pneumothorax	7 (5.8)	8 (7.8)	9 (8.4)	0.73
Bronchopulmonary dysplasia [5] mod/severe	33 (27.5)	17 (16.7)	30 (28.0)	0.09

(Data in each phase is not randomized). Values in cell represent n (% unless stated otherwise). P value < 0.05 is significant. 1- LASER or Anti-Vascular endothelial growth factor, 2- Blood and/or cerebrospinal fluid culture positive for bacteria, 3- intraventricular hemorrhage of ≥ grade 3, periventricular leukomalacia or cerebral hemorrhage, 4- medical or surgical treatment, 5- positive pressure Resp support or supplemental oxygen at 36 weeks post menstrual age
